# Natural polysaccharides exert anti-tumor effects as dendritic cell immune enhancers

**DOI:** 10.3389/fonc.2023.1274048

**Published:** 2023-10-09

**Authors:** Hongtai Xiong, Xinpu Han, Liu Cai, Honggang Zheng

**Affiliations:** ^1^ Department of Oncology, Guang’anmen Hospital, China Academy of Chinese Medical Sciences, Beijing, China; ^2^ The First Clinical Medical College, Shaanxi University of Chinese Medicine, Xianyang, China

**Keywords:** natural polysaccharide, dendritic cell, anti-tumor mechanism, immune function, DC vaccine, nanoparticle

## Abstract

With the development of immunotherapy, the process of tumor treatment is also moving forward. Polysaccharides are biological response modifiers widely found in plants, animals, fungi, and algae and are mainly composed of monosaccharides covalently linked by glycosidic bonds. For a long time, polysaccharides have been widely used clinically to enhance the body’s immunity. However, their mechanisms of action in tumor immunotherapy have not been thoroughly explored. Dendritic cells (DCs) are a heterogeneous population of antigen presenting cells (APCs) that play a crucial role in the regulation and maintenance of the immune response. There is growing evidence that polysaccharides can enhance the essential functions of DCs to intervene the immune response. This paper describes the research progress on the anti-tumor immune effects of natural polysaccharides on DCs. These studies show that polysaccharides can act on pattern recognition receptors (PRRs) on the surface of DCs and activate phosphatidylinositol 3-kinase (PI3K)/protein kinase B (AKT), mitogen-activated protein kinase (MAPK), nuclear factor-κB (NF-κB), Dectin-1/Syk, and other signalling pathways, thereby promoting the main functions of DCs such as maturation, metabolism, antigen uptake and presentation, and activation of T cells, and then play an anti-tumor role. In addition, the application of polysaccharides as adjuvants for DC vaccines, in combination with adoptive immunotherapy and immune checkpoint inhibitors (ICIs), as well as their co-assembly with nanoparticles (NPs) into nano drug delivery systems is also introduced. These results reveal the biological effects of polysaccharides, provide a new perspective for the anti-tumor immunopharmacological research of natural polysaccharides, and provide helpful information for guiding polysaccharides as complementary medicines in cancer immunotherapy.

## 1 Introduction

Tumor is one of the major health threats to human health and life worldwide. Previous treatments for tumor such as surgery, chemotherapy, and radiotherapy, all directly target the tumor itself, which can cause damage to normal cells of the body while playing a therapeutic role (1). In recent years, tumor immunotherapy has emerged as a frontier in the clinical treatment of tumors by enhancing the activity of the body’s immune system to kill tumor cells (2). At present, immunotherapies that have been successfully approved for clinical use include oncolytic viruses, cancer vaccines, immune checkpoint inhibitors (ICIs), and adoptive cell therapy (3). Oncolytic viruses act by directly lysing tumor cells and activating innate and adaptive immunity (4). Cancer vaccines have been shown to trigger T cell-mediated anti-tumor immune responses using tumor-associated antigens (TAAs) (5). In response to suppressive ligands such as programmed cell death-ligand 1 (PD-L1), which are highly expressed in the tumor microenvironment (TME), inhibiting the function of lymphocytes, scientists have invented ICIs to treat tumors (6). Adoptive cell therapy involves isolating autoimmune cells, expanding them *in vitro*, and reinjecting them into the patient to eliminate cancer cells (7). More recently, researchers have combined immunotherapy with other strategies to achieve better anti-tumor results. Zhang et al. (8) found that Glucose oxidase (GOx)-Mn nanoparticles (NPs) could consume glucose and increase tumor glycolysis activity, and combined with ICIs had a good therapeutic effect on both B16F10 tumor and 4T1 tumor. Chen et al. (9) found that cryoablation can promote the release of TAAs into the blood, and prolong the survival of patients with hepatocellular carcinoma after combined with allogeneic NK cell infusion. In addition, advances in bioinformatics have also advanced the application of immunotherapy. Single-cell techniques, such as mass cytometry and single-cell RNA sequencing, are able to systematically characterize the cell populations in the TME (10). The design of tissue engineering and microphysiological models allows rapid evaluation of novel cell-based immunotherapeutic strategies for solid tumors (11). Morazán-Fernández et al. (12) developed an in silico pipeline to predict antigens present in distinct locations of the tumor, with potential use for developing immunotherapies for some specific types of cancer. Although immunotherapy has made impressive progress, obstacles and challenges, including limited response rates, inability to predict clinical efficacy, and the emergence of drug resistance and related side effects, have prevented further clinical use of immunotherapy (13). Therefore, continuing to explore ways to improve the effect of immunotherapy is an inevitable trend of tumor treatment.

Dendritic cells (DCs) are the most functional antigen presenting cells (APCs) identified *in vivo*. As sentinels of immune cells, the cellular immunological response triggered by DCs is crucial for the body’s anti-tumor immunity (14). DCs have a crucial role in regulating both innate and acquired immune responses, and in innate immunity are able to identify and respond to pathogen associated molecular patterns (PAMPs) or damage associated molecular patterns (DAMPs) generated after cell death (15). In adaptive immunity DCs can convert proteins into peptides presented on major histocompatibility complex (MHC) molecules by intracellular enzymatic system, which are further presented to naive T cells to trigger the immune response of T cells (16). Currently, an increasing number of researchers are inclined to regulate DCs to more effectively activate and drive T cells into the TME, especially for those cold tumors with weak immunogenicity, in order to improve the efficacy of immune checkpoint therapy (17).

It is a hotspot in the research of anticancer drugs to find superior anti-tumor active substances from natural resources. Baicalin, a flavonoid compound in Scutellaria baicalensis, which inhibits epithelial mesenchymal transition by affecting proteins such as TGF-B/Smad, Ezrin (18). Triterpenes in Ganoderma lucidum, which can directly inhibit the proliferation of the gastric carcinoma SGC-7901, the lung carcinoma A549, and the lymphoma Ramos (19). Astragalus saponins can inhibit tumor angiogenesis by regulating mTOR signalling and decreasing the level of VEGF protein expression in tumor cells (20). Compared with other natural compounds such as flavonoids, terpenes and saponins, polysaccharides have the characteristics of wide source, strong pharmacological activity, abundant target and high safety (21). Polysaccharides are mainly composed of monosaccharides covalently linked by α- or β-glycosidic bonds. According to different sources, polysaccharides can be divided into five categories: fungal polysaccharides, plant polysaccharides, lichen and algal polysaccharides, animal polysaccharides, and bacterial polysaccharides (22). Due to the complex branching structure and highly heterogeneous sugar composition of polysaccharides, different polysaccharides exhibit highly heterogeneous biological activities (23). Plants are an important source of natural polysaccharides, which can be divided into intracellular polysaccharides (mainly fructosan and mannan), cell wall polysaccharides (mainly cellulose) and extracellular polysaccharides (mainly galactosan and glucuronic acid) according to their presence in plant cells (24). The glycosidic bonds of plant polysaccharides are mainly α-(1 → 6)-D, α-(1 → 4)-D, and β-(1 → 4)-D. It was found that most of the plant polysaccharides connected with α-(1 → 6)-D glucoside bond had high anti-tumor, anti-virus, anti-radiation and anti-coagulation activities (25). Lo et al. (26) proposed that plant polysaccharides composed of arabinose, mannose, xylose and galactose could promote macrophage activation. One of the most abundant forms of polysaccharide found in bacteria and fungi (27), β-glucan is able to act on several immune receptors, including Dectin-1, complement receptor, activating a range of immune cells and exerting potent anti-tumor effects (28). Unlike β-glucan, α-glucan is predominantly found in the cell walls of fungi (29), but due to its insolubility in water and rarely specifically recognised by immune receptors, α-glucan does not have high biological activity (30). Animal polysaccharides are widely found in animal connective tissues, commonly chitin, heparin, chondroitin sulphate, hyaluronic acid, keratan sulphate, and have good biocompatibility with human cells (31). Polysaccharides extracted from marine organisms, such as shark chondroitin, have strong anti-tumor, anti-aging and bone-protecting activities (32). In conclusion, polysaccharides play an important role in the treatment of many diseases, especially tumors. In recent years, data from numerous experiments have proved that in the TME, the anti-tumor effect of polysaccharides is not only achieved through direct induction of apoptosis in tumor cells but is also thought to be related to the upregulation of immune response, especially the regulation of DCs’ maturation and function, which can significantly affect the host immune system and stimulate immune cells’ anti-tumor activity (33, 34). This paper provides an overview of the research progress on the anti-tumor immunological effects of natural polysaccharides on DCs, with a view to providing new perspectives on the anti-tumor immunopharmacological research of natural polysaccharides.

## 2 DCs and tumor immunity

### 2.1 Classification and origin of DCs

**Figure 1:**
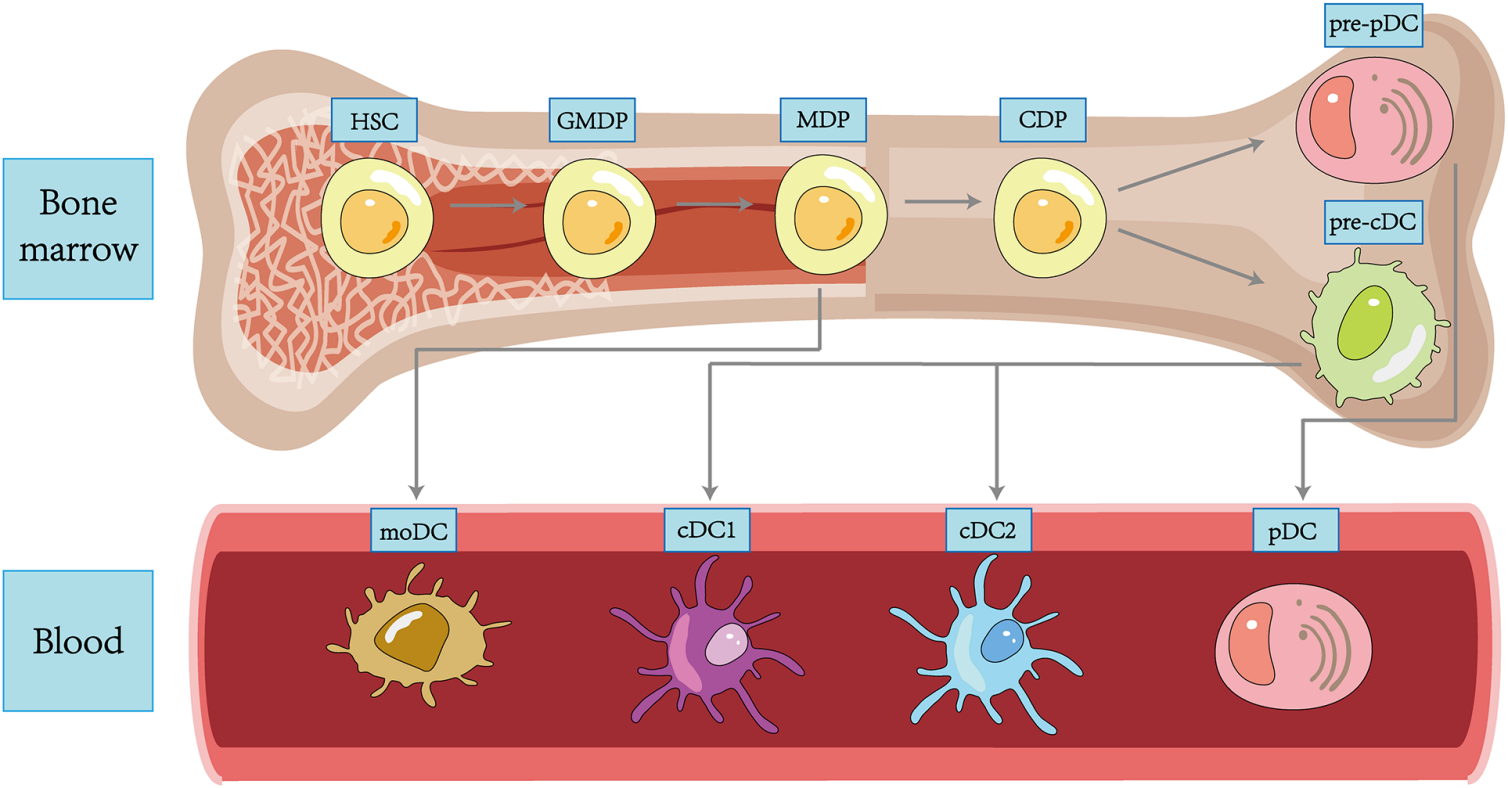
Ontogeny and differentiation of DCs within tissues.

In 1973, Ralph Steinman and Zan Cohn identified a population of cells with a distinctive star-shaped morphology from mouse spleens and named them DCs. Since then, more and more subpopulations of DC have been revealed in the course of research (35), and transcriptional analysis also demonstrated that each DC subpopulation had a conserved gene expression profile across tissues and species (36). DC subpopulations are currently classified using an integrated methodology considering different factors such as origin and differentiation pathways, developmental stages, key genetic features, Toll-like receptors (TLRs), and other functionally relevant molecules (37). We broadly classify DCs into plasmacytoid DCs (pDCs), type 1 classical DCs (cDC1s), type 2 classical DCs (cDC2s), and monocyte-derived DCs (moDCs) (38) (**Figure 1**).

Although the upstream precursors of DCs have been debated for many years, it is generally accepted that DCs originate from hematopoietic stem cells (HSCs) and complete precursor differentiation from HSCs to DC progenitors (GMDPs) to monocyte and DC progenitors (MDPs) in the bone marrow (39). A proportion of MDPs further differentiate into common DC progenitors (CDPs), which differentiate into classical DC precursors (pre-cDCs) and plasmacytoid DC precursors (pre-pDCs), with the differentiation regulated by Fms-like tyrosine kinase 3 ligand (FLT3L) (14). pDCs are differentiated in the bone marrow, regulated by transcription factor E2-2, and subsequently migrate through the circulation to both lymphoid (like lymph nodes and spleen) and non-lymphoid tissues (like lung, skin, and gastrointestinal tract) tissues (40). In the unstimulated physiological state, pDCs express low levels of MHC-II and costimulatory molecules, as well as small amounts of pattern recognition receptors (PRRs), including TLR7 and TLR9, upon recognizing exotic nucleic acids, they produce high levels of IFN-I and develop the capacity to present foreign antigens (41). Both classic DCs are differentiated from pre-cDCs in peripheral lymphoid tissue. The development of cDC1s depends on the transcriptional factor BATF3. cDC1s can efficiently cross-present antigens through MHC-I and activate CD8+ T cells while enhancing the immune effect of type 1 helper T (Th1) cells and natural killer (NK) cells via IL-12 (42). cDC2s are present in blood, lymphoid organs, and tissues, including the spleen, kidney, dermis, and intestine, and are highly heterogeneous, with development regulated by a variety of transcription factors, including IRF-4 and ZEB-2 (43). Compared to cDC1s, they have a more extraordinary ability to synthesize IL-12, activate Th1, Th2, and Th17 cells *in vitro* via MHC-II, and have a broad immune response (44). A further proportion of MDPs will develop into monocytes, which can differentiate into moDCs and function as cDCs in pathogen-induced inflammation (45). In addition, Langerhans cells (LCs) belong to a specific group of resident APCs in the skin epidermis that present antigen to CD4+ T cells to induce a Th2-type immune response (46). Most of the literature previously considered LCs to be a class of DCs. However, LCs are clearly distinct from DCs in terms of individual development and are capable of self-renewal (47). In addition, LCs are developmentally dependent on the macrophage colony stimulating factor receptor (M-CSF-R) and IL-34 and not on FLT3L. Based on these characteristics, LCs have been grouped into a macrophage population in some recent reports (48).

### 2.2 Activation and anti-tumor response of DCs

In the absence of pathogen recognition or exposure to inflammatory cytokines, heterogeneous populations of immature DCs with sentinel function exist in the first line of organismal defense, such as skin, digestive system, and respiratory mucosa. DCs in this state have a strong antigen capture and processing function, capturing invading antigens through their specific endocytic system and a large number of uptake receptors (49, 50). If the PRRs on DCs recognize TAAs, then DCs shift gene transcription and cellular metabolism, gradually migrating to lymphoid organs and transforming into mature DCs (51). The morphological features are altered, with an enlarged cytoplasm accompanied by more dendritic protrusions. Phenotypically, DCs show increased MHC class and costimulatory molecules expression on their surface (52). The upregulation of costimulatory molecules can provide signals required for T cell activation, e.g., CD80 and CD86 expressed by DCs regulate T cell activation by interacting with CD28 or CTLA4, and the interaction of CD40 with CD40L is critical for the function of CD4+T cells (53). Mature DCs expressing CCR7 migrate to the T cell compartments of secondary lymphoid tissues and initiate a specific cellular immune response through MHC-I restricted cytotoxic T lymphocytes (CTLs) response and MHC-II restricted CD4+ T cell response (54). The activated effector T cells then move to the tumor target site and specifically identify tumor cells through T cell receptor and MHC interactions. After killing the tumor cells, the effector T cells promote the release of TAAs, which in turn promotes a virtuous cycle of tumor immunity and a sustained anti-tumor effect (55).

Recent studies have found that the innate immune response for tumors is mainly caused by the stimulator of interferon genes (STING) pathway (56). The replacement and death of tumor cells may lead to the release of their own DNA and other DAMPs into the infiltrating DCs, recognized by DCs cytoplasmic DNA receptors such as cyclic guanosine monophosphateadenosine monophosphate synthase (cGAS), which catalyzes the ligation of two nucleotides to form cyclic dinucleotides (CDNs). CDNs bind to STING as second messengers and activate it. After STING activation, downstream TBK1 and IRF3 are recruited to promote the maturation of DCs to achieve antigen presentation. Thus, the innate immune response was linked to the adaptive immune response (57). In addition, DCs can also influence tumor progression by secreting chemokines and cytokines. When activated, TLR7 and TLR9 on pDCs can secrete large amounts of IFN-I, which can both inhibit the proliferation of tumor cells and activate the immune system to exert anti-tumor effects (58). Both cDC1s and cDC2s can produce cytokines such as IL-12, IL-18, and IL-23 upon stimulation of TLRs, which can promote NK cell activation and exert effector functions (59). DCs can also produce chemokines that recruit T cells in TME. For example, cDC1s are the main secretory cells of C-X-C motif chemokine ligand 9 (CXCL9) and CXCL10 in TME, which promote infiltration of CD8+ T cells into the TME and maintain effector T cells at the tumor site for long periods (60).

## 3 Effect of TME on DCs

TME is the environment in which tumor develops and consists of tumor cells, fibroblasts, endothelial cells, infiltrating immune cells, and extracellular matrix components. Tumor cells are extremely capable of growth and can use a variety of nutrients to adapt to changing environmental conditions, resulting in a combination of abnormal responses such as hypoxia, disrupted glycolysis, lipid accumulation, and ferroptosis, ultimately inhibiting the host immune system’s response to DCs (61).

Tumor cells need to induce angiogenesis to meet their oxygen and nutrient requirements. Abnormal tumor vasculatures lead to the creation of hyperpermeable, dilated, and gyral occlusive areas in the TME, resulting in poor blood perfusion that cannot meet the needs of the tumor, resulting in visual hypoxia (62). Yang et al.(63) used microarray analysis to examine the transcriptional patterns of human moDCs grown in normoxia and hypoxia. They found that hypoxic DCs were significantly less able to induce T cells proliferation compared to normoxic DCs. Han et al.(64) further investigated the mechanism by which the hypoxic environment inhibits DCs and found that it may be related to vascular endothelial growth factor (VEGF) induced by hypoxia induction factor (HIF). HIF is one of the essential molecules that express hypoxia signalling in the TME, which can directly activate the transcription of VEGF through the binding of hormone response elements. VEGF can bind to different receptors and perform different functions, among which VEGFR-3 can block TLR4/nuclear factor-κB (NF-κB) activation, thereby inhibiting the translocation of DCs to draining lymph nodes. Related studies have also found that VEGF is the main cause of direct inhibition of maturation of DCs derived from CD34 precursors in most solid tumors (65). Hypoxia can also affect DCs through the production of reactive oxygen species (ROS). In hypoxia, cells produce ATP through glycolysis, which increases the level of ROS in tumor cells and immune cells (66). High levels of ROS can enter DCs by diffusion across the plasma membrane or through extracellular vesicles released by tumor cells, affecting the oxidation of high mobility group protein B1 (HMGB1), thereby preventing the maturation of DCs and affecting their antigen-presenting function (67).

Upon activation, immune cells undergo metabolic reprogramming from oxidative phosphorylation to aerobic glycolysis (68), which is necessary for the membrane integrity, energy production, and migratory capacity of activated DCs. The loading of peptides onto MHC-II and the expression of costimulatory molecules such as CD80 and CD86 on the surface of DCs also require ATP produced by glycolysis (69). Disturbed vascular transport within tumor tissue allows most of the glucose to be absorbed by tumor cells, resulting in a glucose-deprived TME that leads to a weakened glycolytic response to meet the high energy demands required for DCs to mature (70). *In vitro*, glucose is also essential for the migration of DCs to CCL21, and studies have shown that blocking glycolysis reduces the migratory capacity of DCs (71).

The growth of tumor has exceptionally high requirements for energy. Fatty acids, as crucial energy sources and essential components of cell membranes, are closely related to cell proliferation in the TME, and increased lipid uptake is also considered a sign of malignant tumors (72). Herber et al. (73) observed DCs under a normal lipid environment and DCs under a high lipid environment by flow cytometry and found that DCs’ ability to process antigens under high lipid environment was severely reduced. Gao et al. (74) upregulated the levels of lipoprotein lipase and triacylglycerol of thymic lymphoma mice through whole-body irradiation and found that DCs surface marker molecules were significantly downregulated in the high lipid environment and DCs could not effectively stimulate T cells to perform the immune function.

Ferroptosis is a newly discovered type of programmed cell death after apoptosis, necrosis, pyrodeath and autophagy death (75). The iron content in normal cells is in steady state, and the imbalance of iron circulation in tumor cells can easily lead to excess iron and mediate excessive accumulation of ROS. When the ROS clearance function of glutathione peroxidase 4 (GPX4) is weakened, cell membrane damage is induced through iron-dependent phospholipid peroxidation process, thus inducing cell death (76). Wiernicki et al. (77) found that co-incubation with GPX4-depleted ferroptosis cancer cells had a negative impact on the maturity level of bone marroe-derived DCs (BMDCs), which was manifested by the decline of CD86, CD40, and MHC-II and the production of inflammatory factors such as IL-6, IL-12, TNF, and IFN-β. This may be related to the fact that ferroptosis does not evoke the exposure of phosphatidylserine at the outer leaflet of the plasma membrane prior to cell membrane permeabilization, resulting in the disappearance of the “eat me” signal.

## 4 Anti-tumor mechanism of natural polysaccharides acting on DCs

**Figure 2:**
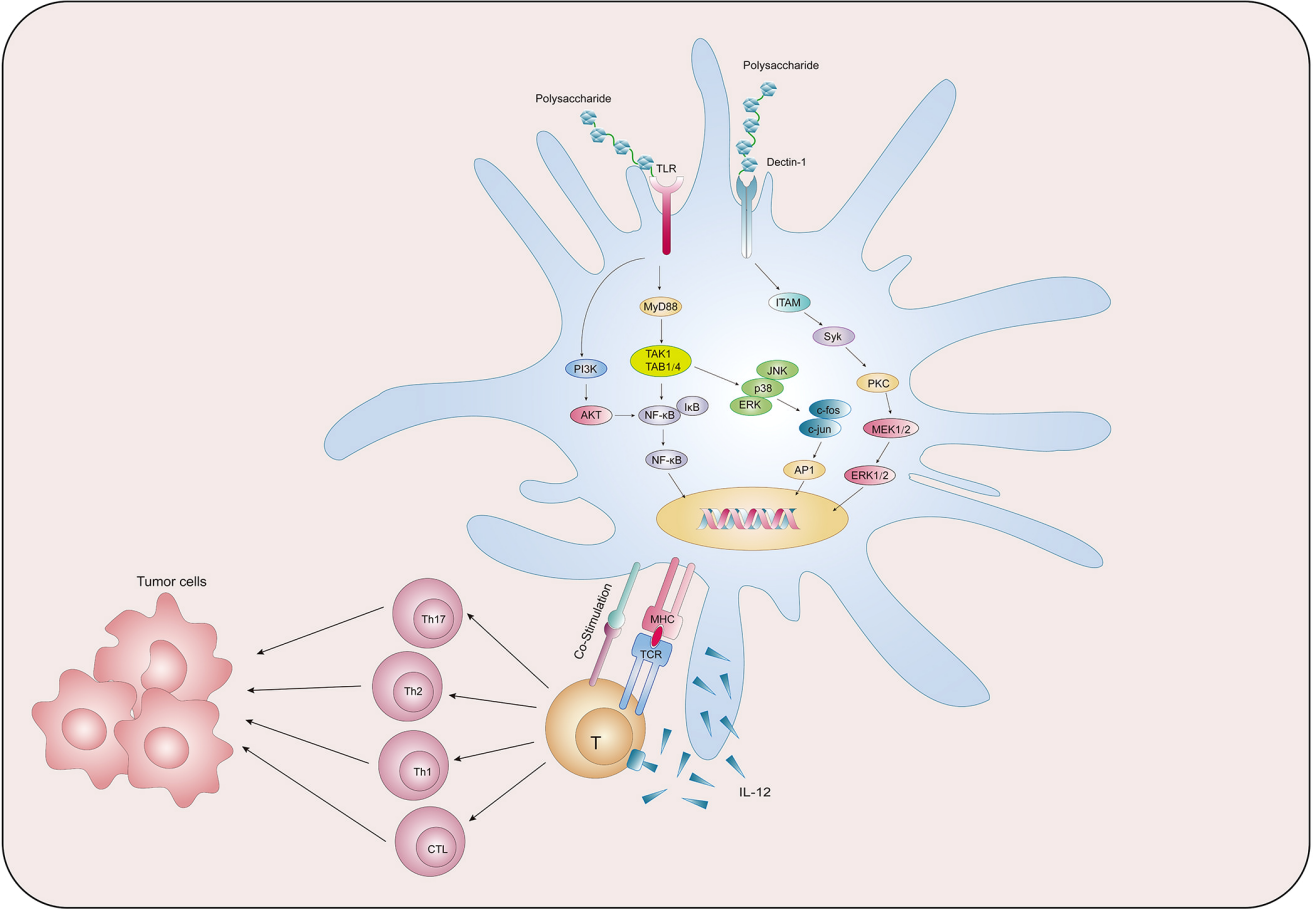
Natural polysaccharides act on DC to exert anti-tumor effects.

We collected existing research data and found that natural polysaccharides could act on PRRs on the surface of DCs and activate multiple signalling pathways, thereby promoting the main functions of DCs such as maturation, metabolism, antigen uptake and presentation, and activation of T cells, so as to exert anti-tumor function (**Figure 2**). **Table 1** summarises the findings.

**Table 1:** Summary of the mechanisms of DC activation by natural polysaccharides.

Name	Phenotypic maturation	Membrane target	Downstream signalling	T cell activation	Cytokine production	Anti-tumor effect	References
POL-P3b	↑CD40, CD80, CD86, MHC-II	TLR4	NF-κB	↑proliferation of CD4+ and CD8+ T cells and CD4+/CD8+	↑IFN-γ, IL-12p70, TNF-α, and IL-4	POL-P3b-treated DC vaccine can improve tumor inhibition rate and inhibit lung metastasis	(78)
APS	↑CD206, CD80, CD86	Not tested	NF-κB	↑proliferation of CD45+CD8+ T cells	Not tested	APS suppressed tumorigenicity and metastasis in syngeneic C57BL/6 mice, and potentiated anticancer effect of cisplatin *In vivo*	(79)
LNT	↑CD86	Not tested	Not tested	Not tested	↑IL-6, IL-10, and TGF-β1	The survival period of Colon-26-bearing mice treated with S-1 + LNT was significantly more prolonged than that of mice treated with S-1 alone	(80)
GLP	↑CD40, CD80, CD86, MHC-II	TLR4	Not tested	↑proliferation of CD4+/CD44+ memory T cells	↑mRNA expression of IL-6, IL-12, IL-1β, TNF-α, and IFN-γ	GLP exhibited strong inhibitory effects on 4T1 tumor growth and pulmonary metastasis when combined with doxorubicin	(81)
NGP	↑CD40, CD80, CD83, CD86, MHC-II	Not tested	Not tested	↑proliferation of T cells	↑IL-12p70, TNF-α	Not tested	(82)
APS-AuNP	↑CD40, CD80, CD86, MHC-II	Not tested	Not tested	↑proliferation of CD4+ T cells and CD8+ T cells	↑IL-6, IL-1β, IFN-γ, and TNF-α	The inhibitory rate of APS-AuNP against 4T1 primary tumor growth and pulmonary metastasis in mice was higher than paclitaxel-treated group	(83)
POL-P3b	↑CD40, CD80, CD86, MHC-I, MHC-II	TLR4	PI3K/AKT (↑phosphorylation of AKT)	Not tested	Not tested	The tumor weight of U14-bearing mice in the POL-P3b group was significantly lower than that of the model group	(84)
Angelan	↑MHC-I, MHC-II, CD80, CD86	Not tested	MAPK (↑ phosphorylation of ERK, JNK, and p38); NF-κB (↑nuclear translocation of p50, p65, RelB, and c-Rel)	Not tested	↑IL-12, CCL19, CXCL12	Angelan-treated mature DC more effectively inhibited B16F10 tumor growth than immature DCs in syngenic murine tumor model	(85)
AC hmwPS	↑CD40, CD80, CD86, MHC-I, MHC-II	TLR2 and TLR4	MAPK (↑ phosphorylation of ERK, JNK, and p38); NF-κB (↑nuclear translocation of p65)	↑proliferation of CD8+ T cells; ↑Th1 differentiation	↑TNF-α, IL-6, and IL-12	In a mouse tumor model, AC hmwPSs enhanced the anti-tumor efficacy of the HER-2/neu DNA vaccine by facilitating specific Th1 responses	(86)
YM-2A	↑CD80, CD86	Dectin-1	Dectin-1-dependent pathway	↑proliferation of CD4+ and CD8+ T cells	↑IFN-γ	YM-2A-treated TAA-loaded DC vaccine significantly reduced tumor growth and improved survival in two murine tumor models, CT-26 tumor-bearing BALB/c mice and B16 melanoma-bearing C57BL/6 mice	(87)
LBP	↑CD80, CD86	Not tested	↑expression of Notch, Jagged, Hes1, and Hes5	↑proportion of CD3+CD8+ T cells	↑IL-12; ↓IL-10, TGF-β	Administration of LBP Strengthened the Cytotoxicity of DC-Mediated CTLs on Colon Cancer Cell CT26-WT	(88)
PLP	↑CD80, CD86, MHC-II	Not tested	Not tested	↑proportion of CD3+CD8+ T cells	↑IL-12p70, TNF-α, IL-1β, and IL-6	TAXOL+PLP significantly inhibited 4T1 tumor growth, compared with the control group and TAXOL group	(89)
RGP	↑CD40, CD80, CD86, MHC-I, MHC-II	TLR4	Not tested	↑proportion of OT-I and OT-II T cells	↑IL-6, IL-12p40, TNF-α	The combination of RGP and Ag effectively inhibited the growth of CT57 tumors and B6 melanoma in BLAB/c and C26BL/16 mice	(90)
GLP	↑CD40, CD80, CD86, and MHC-II	Not tested	Not tested	↑proportion of CD3+CD8+ T cells	↑IL-10 and IL-12p70	GLP significantly reduced the tumor volume of glioma-bearing rats to 101.93 ± 53.58 mm^3^ and 113.56 ± 39.76 mm^3^, compared to 162.99 ± 48.34 mm^3^ in the control group	(91)
PS-F2	↑CD40, CD80, CD86, and MHC-II	Not tested	Not tested	↑Th1 differentiation	↑TNF-α, IL-12/IL-23 p40, IL-6, and IL-10	Mice immunized with PS-F2 and OVA were almost completely protected from MO5 tumor challenge	(92)

↑, increases; ↓, decreases.

### 4.1 Phenotypic maturation

DCs highly express MHC class molecules, which recognize tumor antigens and form peptide-MHC class molecular complexes that are ultimately presented to T cells to initiate the immune response. In addition, DCs highly express costimulatory molecules, which provide a second signalling molecule for T cell activation (93). Thus, MCH class and costimulatory molecules can be used as classical phenotypic markers of DCs maturation.

Jia et al. (78) used Portulaca oleracea L. polysaccharide (POL-P3b) to interfere with BMDCs and found that POL-P3b could increase the expression of CD40, CD80, CD86, and MHC-II by promote DCs maturation, as evidenced by rougher cell surfaces, folds, and elongated dendrites. The authors then transfused POL-P3b-sensitized DCs into breast cancer mice and found that the tumor inhibition rate increased from 37.46% to 50.79% compared with the non-sensitized DCs group, suggesting that POL-P3b plays an anti-tumor role by promoting the maturation of the DCs phenotype. Bamodu et al. (79) found that Astragalus polysaccharide (APS) was able to disrupt tumorspheres formed by H1299 cells, a type of human-derived lung cancer cell, in a dose-dependent manner, and furthermore, APS combined with cisplatin resulted in a 74.5% reduction in tumor size and a 91.8% reduction in the number of metastatic nodules in LLC1 tumor-bearing C57BL/6 mice. To understand the basis of the action of APS, the authors used APS in combination with GM-CSF and IL-4 to induce the differentiation of isolated peripheral blood mononuclear cells (PBMCs) into DCs in lung cancer patients and found that the GM-CSF+IL-4 combined with APS group induced more CD80+ (16%), CD103+ (15%) and CD86+ (13%) cells production compared to the GM-CSF+IL-4 group alone. Mushiake et al. (80) found that the anticancer drug S-1, combined with lentinan (LNT), was able to prolong the survival of Sato lung carcinoma–bearing rats from 11.1 ± 0.7 to 15.4 ± 2.1 days, and immunohistochemical staining showed that the accumulation of tumor-infiltrating CD86+ DCs in the combination group was twice as much as that of the non-drug group.

### 4.2 Antigen phagocytosis

Immature DCs have high antigen capture and phagocytosis activity, which gradually decreases during the maturation of DCs so that the antigen uptake capacity can reflect the maturity of DCs to a certain extent (94). One study (81) found that the combination of Ganoderma lucidum polysaccharide (GLP) and doxorubicin (DOX) could effectively alleviate the increase in tumor volume in mice with breast cancer. Compared to mice treated with PBS that showed a tumor volume of approximately 1500 mm^3^ on day 20, the tumor volume in the GLP+DOX-treated group was only 620 mm^3^, and H&E staining of tumor tissues revealed that the cells in the GLP+DOX-treated group were significantly shrivelled and the nuclear of the cells were fragmented. Next, the authors incubated DCs with FITC-Dextran labeled antigen particles after 24 hours of GLP intervention and then used flow cytometry to detect the mean fluorescence intensity of DCs expression. It found that compared with the control group, all concentrations of GLP (10ug/ml, 20ug/ml, 40ug/ml) reduced the absorption of FITC-Dextran by DCs. Pang et al. (83) used immunoactive polysaccharide functionalized gold nanocomposites (APS-AuNP) to stimulate DCs *in vitro* and found that APS-AuNP reduced the uptake of FITC-Dextran by DCs in a dose-dependent manner. The authors then measured its inhibitory effect on primary and metastatic tumors in mouse models of breast cancer, and found that APS-AuNP had a higher inhibitory rate on 4T1 primary tumor growth and lung metastasis of mice than the paclitaxel treatment group.

Acid phosphatase (ACP) is a key enzyme in the degradation of intracellular antigens in DCs. When DCs mature, ACP activity decreases due to the completion of the antigen treatment process, and ACP activity measurement helps to detect the phagocytic capacity of DCs (95). Meng et al. (82) used 200 μg/ml Neutral ginseng polysaccharide (NGP) to treat BMDCs for 48 h. The phenol-4-AAP (amino anti-pyrine) method was used to determine the ACP activity in DCs and found that the ACP activity was significantly reduced after NGP intervention, signifying the gradual termination of phagocytosis and antigen uptake of DCs. Meng’s team also used transmission electron microscopy to observe the internal cytoplasm and organelles of DCs and found that DCs contained fewer lysosomes and vesicles after NGP intervention, corroborating the experimental results that NGP triggered a reduction in antigen phagocytosis in DCs.

### 4.3 Natural polysaccharides pulsed DCs-based anti-tumor immunity through various signaling pathways

PRRs, including TLRs, C-type lectin receptors (CLRs), and cytoplasmic sensors, are present on the cell membrane surface of DCs. DCs are able to use PRRs to sense PAMPs or DAMPs carried by pathogens from the intracellular and extracellular environments and initiate an adaptive immune response (96). TLR, named for its structural similarity to Toll protein in Drosophila, contains abundant leucine repeats and is the most important class I transmembrane protein receptor for sensing pathogens in the environment (97). After binding with PAMPs, TLR can activate the signaling pathway of phosphatidylinositol 3-kinase (PI3K)/protein kinase B (AKT), mitogen-activated protein kinase (MAPK), and NF-κB through the key protein myeloid differentiation factor 88 (MyD88), thus generating a series of immune responses (98). Zhao et al. (84) established a cervical cancer model by subcutaneously injecting U14 cells into mice, and then administrated POL-P3b intragastrically, and found that different doses of POL-P3b significantly inhibited tumor growth, among which, the tumor inhibition rate of the high-dose group reached 46.56%. The excellent anti-tumor effect was related to the protection of intestinal DCs from apoptosis by POL-P3b. Afterwards, the authors discussed more detailed mechanisms and found that POL-P3b treatment increased the TLR4 level as well as the phosphorylation of AKT. After applying the PI3K kinase inhibitor LY294002 to inhibit the activity of AKT in intestinal DCs, POL-P3b further inhibited AKT activity, indicating that POL-P3b exerts anti-tumor effects by activating the TLR4-PI3K/AKT signalling pathway within DCs to exert anti-tumor effects. Kim et al. (85) found that DCs treated with Angelan, an acidic polysaccharide isolated from Angelica sinensis, inhibited the growth of B16F10 melanoma cells by 69%, while control DCs inhibited tumor growth by 49%. The authors (99) studied the relevant mechanism in more detail, and found that Angelan could increase the surface expression of MHC-II, CD80 and CD86 molecules in DCs of C3H/HeN(TLR4+/+) mice, but had no significant effect on DCs of C3H/HEJ(TLR4-/-) mice, and furthermore, Angelan increased nuclear translocation of NF-κB p50, p65, RelB and c-Rel, and phosphorylated ERK, JNK and p38 MAPKs, suggesting that Angelan induces maturation of DCs through the TLR4-NF-κB and TLR4-MAPK pathways. Activation of NF-κB and MAPK pathways can also be found in DCs after intervention by Antrodia cinnamomea high-molecular weight polysaccharide (AC hmwPS) (86).

CLR is a large protein superfamily. A typical CLR contains one or more glycobase recognition domains, and two Ca2+ binding sites are present in the ring protruding from the protein surface (100). Dectin-1 is a type II CLR that mediates chemotactic movements, inflammatory factor secretion, and other cellular responses. YM-2A is a polysaccharide isolated from Grifola frondosa, and the researchers (87) found that after injection of YM-2A-treated DCs, tumor growth was significantly inhibited in both CT26 tumor-bearing BALB/c mice and B16 melanoma-bearing C57BL/6 mice. After blocking Dectin-1 with anti-Dectin-1 antibody, the expression of CD80, CD86 and MHC-II in DCs induced by YM-2A was almost completely eliminated, as well as the secretion of IL-12p40, suggesting that YM-2A may induce DCs maturation through Dectin-1/Syk pathway.

As research has progressed, some signalling pathways not dependent on PRRs have also been shown to play a significant part in the activation of DCs. The Notch signalling pathway is involved in the regulation of CD4+ T cells by DCs. It is composed of Notch receptors, ligands, and downstream molecules and transmits biological signals through the interaction of receptors and ligands between neighboring cells (101). Wang et al. (88) co-cultured T cells and DCs isolated from mice with Colon Cancer Cell CT26-WT and then treated with 100ng/ml Lycium barbarum polysaccharide (LBP) for 20h, and found that the total apoptosis rate of CT26-WT cells in the LBP-treated group was significantly increased. Afterwards, to assess the effect of LBP on DCs, Wang et al. incubated LBP with DCs for 48 h and detected Notch signalling pathway-related molecules Notch, Jagged, Hes1, and Hes5 at the mRNA and protein levels, and found that LBP enhanced the expression of all indicators. These molecular assays confirmed that LBP-induced activation of DCs is dependent on the Notch signalling pathway. In addition, morphological and antibody expression assays were able to confirm this conclusion.

### 4.4 Activation of T cells

After capture of TAAs, mature DCs assemble intracellularly into MHC-I antigenic complexes to the cell surface through an antigenic cross-presentation process, and then DCs migrates to secondary lymphatic organs such as tumor draining lymph nodes under the action of chemokines (102). Here, with the involvement of co-stimulatory molecules that mediate intercellular contacts and cytokine production, antigen-specific activation is carried out, inducing the activation of CD8+ T cells and polarising CD4+ T cells towards the Th1 pathway, which ultimately exerts anti-tumor functions (103). Natural polysaccharides are involved in the activation of T cells by DCs.

Gao et al. (89) administered normal saline, Plantain polysaccharide (PLP), TAXOL and TAXOL+PLP to breast cancer mice loaded with 4T1 cells, compared with the control group and TAXOL group, TAXOL+PLP significantly inhibited the growth of 4T1 tumors, and promoted the aggregation of DCs in the groin lymph nodes, spleen and tumors. After that, the authors co-cultured PLP-treated DCs with lymphocytes and found that the number of CD3+CD8+ T lymphocytes was significantly higher in the PLP group than in the control group, indicating that PLP significantly elicited the CTLs response. Xu et al. (90) found that the combination of Rehmannia glutinosa polysaccharide (RGP) and Ag effectively inhibited the growth of CT57 tumors and B6 melanoma in BLAB/c and C26BL/16 mice. RGP induced DCs maturation and activated antigen-specific immune responses in tumor-bearing mice, causing significant increases in CD4+ and CD8+ T cells, increased mRNA levels of the transcription factor T-BET in Th1 cells, and promoted CSFE-labeled OT-I and OT-II T cells to infiltrate the tumor.

With the progress of research, the mechanism of polysaccharides promoting T cell proliferation through DCs has been gradually reported. Wang et al. (91) found that 50 mg/kg and 100 mg/kg GLP significantly reduced the tumor volume of glioma-bearing rats to 101.93 ± 53.58 mm^3^ and 113.56 ± 39.76 mm^3^, compared to 162.99 ± 48.34 mm^3^ in the control group, and GLP significantly increased the intratumoral infiltration of CD8+ T cells. The researchers also found that DCs in the GLP group secreted higher levels of IL-10 and IL-12p70. IL-12 can polarize the immune system into Th1 response, suggesting that GLP may stimulate DCs to produce major Th1-polarizing cytokines, leading to activation and differentiation of naive CD4+ T cells to Th1 cells. Pi et al. (92) also found that PS-F2, a polysaccharide from Ganoderma lucidum, may initiate Th1 polarization by stimulating DCs to produce IL-12p40, in addition, CD44 expression was significantly increased on CD4+ and CD8+ T cells, and activation of T cells protected mice from MO5 tumor cells. One study (99) found that Angelan-treated DCs highly expressed CCR7 and CXCL12, and had a good response to CCL19 and CXCL12, which are key chemokines for DCs lymph node homing. It suggests that Angelan activates the ability of T cells to kill B19F10 tumor cells at least in part by increasing the migration of DC to the draining lymph nodes.

### 4.5 Metabolic regulation

In TME, the immune profile of DCs is altered by the perception and processing of various metabolic cues in order to adapt to the metabolic competition of tumor cells for essential nutrients such as glucose, amino acids and fatty acids (104). Zhang et al. (105) used UPLC-Q-TOF/MS to detect changes in the metabolism of DCs in the LBP group versus the control group and identified four significantly different metabolites, with betaine, hypoxanthine, and DL threonine-beta-methylaspartic acid increased in the LBP group and L-glutamine significantly decreased in the LBP group. Of these metabolites, hypoxanthine has been shown to cause DCs to exhibit mature morphological features, including increased cell volume and nuclear volume, more open chromatin, and more prominent protrusions (106). The ammonia produced by L-glutamine catabolism restricts MHC-II loading and processing of peptides (107). The team then performed further metabolic pathway analysis with MetaboAnalyst v5.0, which showed that these metabolites were involved in a variety of metabolic pathways, including glycerolipid metabolism, oxidation of branched chain fatty acids, aspartate metabolism, glutamate metabolism, and ammonia recycling, most of which have been shown to be closely associated with DCs activation, maturation, and T cell initiation (108, 109).

## 5 Anti-tumor application of natural polysaccharides acting on DCs

### 5.1 Natural polysaccharides as adjuvants to improve efficiency of DC vaccines

**Figure 3:**
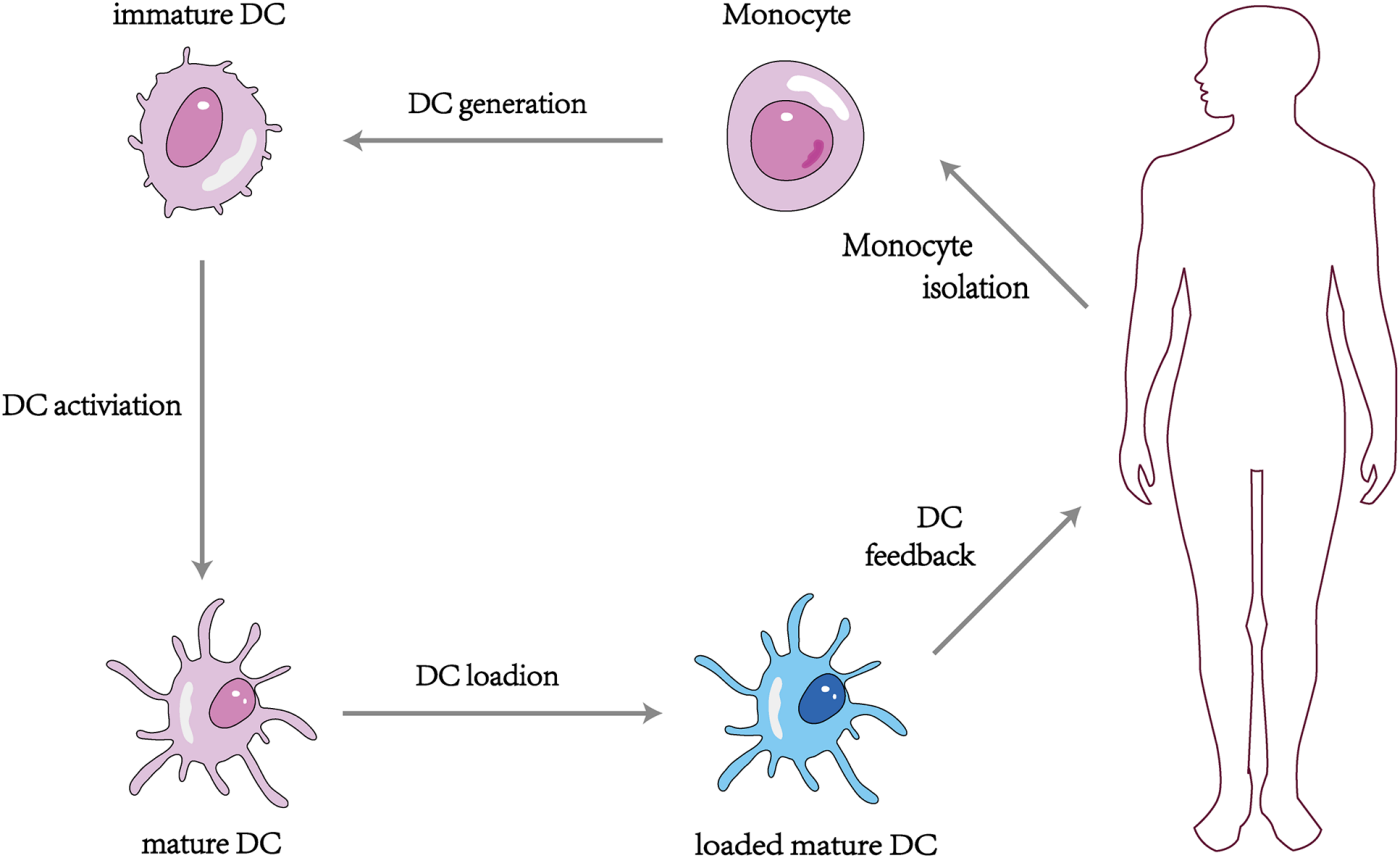
Vaccination strategy with monocyte derived DCs.

The DC vaccine involves obtaining monocytes from the patient’s peripheral blood and differentiating them into DCs using different recombinant cytokines such as rGM-CSF and rIL-4, then loading tumor antigens or antigenic peptides onto the DCs *in vitro* and feeding the antigen-stimulated mature DCs back into the patient’s body to induce the body to produce T lymphocytes with specific killing effects and activate specific cellular and humoral immunity to kill tumor cells (110) (**Figure 3**). At present, more than 200 clinical trials on DC vaccines have been carried out worldwide, and it has been found that DC vaccines can effectively treat ovarian, liver, lung cancer, melanoma, and glioblastoma *in vivo* with a high safety profile (111–113). It was proved that the DC tumor vaccine made by utilizing the important role of DC in activating the body’s immune response is expected to be a breakthrough to conquer tumors (114). However, some clinical application studies of DC vaccines have found limitations in the anti-tumor effects of DC vaccines alone (115), which may be related to the low maturation, poor immune activity, and limited antigen phagocytosis of DC *in vitro* (116).

Based on these influencing factors, polysaccharide combined DC vaccine provides a breakthrough point for establishing a completely new DC vaccine for clinical use (117). Compared with ordinary DC vaccine, the mature DC tumor vaccine induced by polysaccharide as an adjuvant can combine the dual immune effect of polysaccharide and DC, with strong targeting and high stability, and can enhance the killing effect of lymphocytes on tumor cells more effectively. Chang et al. (118) evaluated the efficiency of APS and Codonopsis polysaccharide (CP) to enhance the DC vaccine against 4T1 mammary carcinoma metastasis in mice. They found that DCs treated with APS+CP exhibited the strongest anti-4T1 metastasis activity. The mechanism of action was that the vaccine promoted the expression of the surface molecules CD40, CD80, and CD86 of DCs, allowing DCs to efficiently present antigens to T cells, enhancing the proliferation and activation of CD4+ and CD8+ T cells in mice and delaying tumor growth. A trial (119) investigated the effect of Pleurotus ferulae polysaccharide (PFPS) on the anti-tumor efficacy of a therapeutic HPV DC vaccine. HPV-16 E6 and E7 peptides combined with PFPS were used to stimulate DCs in early (day 5 & 12) and late (day 12 & 19) treatment of tumor mice injected with TC-1 cells. Compared to controls, HPV+PFPS+DCs significantly inhibited tumor growth in both the early and late phases, which was significantly associated with an elevated activation status of CD4+ and CD8+ T cells, suggesting that PFPS may be a potential adjuvant for the DC vaccine. Jin et al. (120) injected OVA+PBS and OVA+Fucoidan into the abdomen of mice, respectively, and found that after OVA+Fucoidan treatment, DCs in the spleen significantly upregulated the expression of CD40, CD80, CD86, and MHC-II, and strongly secreted pro-inflammatory cytokines such as IL-6, IL-12p40, and TNF-α, and promoted Th1 cell response in an IL-12-dependent manner. A permissive transfer assay showed that Fucoidan enhanced antigen presentation and antigen-specific CD4+ and CD8+ T cell activation. The researchers then transplanted B16 tumor cells into both groups of mice and found that the proliferation of B16 cells was significantly inhibited in the mice treated with OVA+Fucoidan.

### 5.2 Natural polysaccharides enhance the effect of adoptive immunotherapy and ICIs

Cytokine-induced killer (CIK) cell is a new type of efficient and immunoactive cell, which is a new choice of adoptive cell immunotherapy for tumor due to its fast proliferation rate, high tumor killing activity, strong cytotoxicity, wide tumor killing spectrum, and low side effects (121). Due to the lack of tumor specificity of CIK cells, pairing DCs with CIK cells has become a new type of immunotherapy, and the combination of DC-CIK cells has shown high anti-tumor activity (122). There is a lot of evidence that DC-CIK cells treated with polysaccharides are more powerful in anti-tumor response than ordinary DC-CIK cells (123). Wang et al. (124) co-cultured APS-induced DCs derived from human monocytes with homologous CIK cells for 15 days to detect the cytotoxic effect of co-cultured cells on esophageal cancer Eca-109 cells, and found that APS could increase the proliferation capacity of DCs and DC-CIK cells. The killing rate of Eca-109 cells in APS-DC-CIK cells group was 75.97 ± 15.74%, which was significantly higher than that in DC-CIK cells group (59.18 ± 7.21%). Zhang et al. (125) found that APS could promote DCs to express CD40, CD80 and HLA-DR molecules. Co-culturing sensitised DCs with CIK cells increased the secretion levels of IL-12 and IFN-γ in the supernatant of DC-CIK cells, and the co-cultured cells more obviously exerted killing effects on A549 cells. Zhou et al. (126) found that Achyranthes bidentata polysaccharide (ABPS) could promote the secretion of IL-12 and TNF-α by DC-CIK cells and enhance the killing activity of DC-CIK cells against the colon cancer cell line SW480.

Tumors activate a series of molecules on immune cells that regulate the level of immune response, known as immune checkpoints, blocking the process of presenting antigens in the tumor immune chain and thus suppressing the immune function of T cells. ICIs can activate immune cell activity by blocking the binding of immune checkpoints to their ligands to achieve anti-tumor effects (127). Cytotoxic T-lymphocyte antigen-4 (CTLA-4) inhibitors and PD-1/PD-L1 inhibitors are two commonly used ICIs. However, the presence of immunosuppressive microenvironment in tumor limits the application of ICIs. Polysaccharides have been found to act as a potential adjuvant in the immunotherapy of ICIs in order to promote the infiltration of immune cells and to improve the therapeutic efficacy (128). Hwang et al. (129) found that intranasal administration of APS can up-regulate the expression of CCR7, increase the number of DCs in mesenteric lymph nodes, promote the expression of CD40, CD80, CD86, MHC I and MHC II in DCs, and then activate NK cells, CD8+ T cells and CD4+ T cells. Later, APS combined with anti-PD-L1 antibody was injected into mice loaded with B16 melanoma cells, and it was found that APS combined with anti-PD-L1 antibody could significantly inhibit lung invasion of B16 cells and prolong the survival of mice to 30 days. These results suggested that APS can be used as an adjuvant to enhance the anti-cancer effects of ICIs.

### 5.3 Nano drug delivery system supports the effect of natural polysaccharides

Although polysaccharides have excellent immunostimulatory activity, their rapid degradation *in vivo* leads to a short duration of action and reduced bioavailability (130). NPs refer to materials that are in the nanoscale range (1 to 100 nm) in at least one dimension in three-dimensional space or are composed of this as the basic unit, including liposomes, solid lipid NPs, polymer micelles, mesoporous silicone NPs, and metallic NPs (131). The inherent small size and high surface area to volume ratio of NPs allow them to bind, absorb and deliver small molecules efficiently. Their variable size, shape, and surface characteristics also give them high stability, high bearing capacity, the property of binding hydrophilic or hydrophobic substances, and compatibility with different routes of administration (132). In recent years, more and more attention has been paid to NPs, which combine polysaccharides with NPs by encapsulation, encapsulation, adsorption or covalent bonding to form “nano drug delivery systems” that can carry polysaccharides directly to tumors and lymphoid organs and provide adequate protection for polysaccharides, promote uptake of DCs and enhance immune response (133, 134). Devi et al. (135) found that NPs conjugated polysaccharides can activate the immune system, allowing it to further act on tumor cells and lead to tumor cell lysis. The controlled release of polysaccharides at the site of action can also be achieved by adding environmental response elements into the NPs (136). In addition, animal cell membranes, endogenous proteins and exosomes are used as carrier materials to encapsulate or modify polysaccharide components to form bionic nano delivery systems, which act as pseudo-endogenous substances and can avoid the rapid metabolism of polysaccharides.

Zhang et al. (81) found that gold nanocomposites containing GLP (GLP-Au) were potent in stimulating DCs for cancer immunotherapy compared to free GLP. Studies co-culturing DCs with T cells demonstrated that GLP-Au-treated DCs significantly increased CD4+ or CD8+ T cell proliferation. When combined with adriamycin, GLP-Au exhibited a strong inhibitory effect on 4T1 tumor growth and lung metastasis. Pang et al. (83) prepared APS-AuNP which were loaded with and presented APS on the surface of AuNP in order to promote a direct interaction between APS and TLR4. APS-AuNP was found to induce maturation of DCs through phenotypic markers with functional changes, promote proliferation of T cells, increase CD4+/CD8+ T lymphocytes as well as effector memory cells, and enhance killing of 4T1 tumor cells as assessed by flow cytometry.The inhibition rate of APS-AuNP on primary tumor growth and lung metastasis of 4T1 in mice was higher than that in paclitaxel-treated group.

Despite the DC vaccine using polysaccharide as an adjuvant, which is expected to become a breakthrough in treating tumor today, inefficient delivery of antigen and adjuvant to secondary lymphoid organs often leads to poor immune response (137). Therefore, combining NP technology with tumor vaccine, encapsulating polysaccharide adjuvant and antigen in the same nanocarrier, and accurately delivering both to DCs can effectively avoid immune tolerance in DCs that ingest antigens without adjuvant (138). This increased efficiency of action is made possible by the high surface area to volume ratio of NPs, which enables efficient loading of tumor antigens and provides sufficient amounts of antigens to stimulate DCs (139). Further active targeting of specific immune cell subsets can be achieved by modifying antibodies or other targeting ligands on the surface of NPs, resulting in more effective antigen stimulation (140). Poly (D, L-lactide-co-glycolide) (PLGA) is a material with excellent biocompatibility and degradability. NPs made from PLGA are highly effective in co-encapsulating antigens and adjuvants and selectively delivering them to DCs (141). Xiong et al. (142) constructed multi-functional PEGylated PLGA NPs encapsulated with APS using a double-emulsion method, which could significantly promote the maturation of DCs and play an important role in initiating adaptive immunity. It was also able to enhance Focused ultrasound-induced immune effects for systemic and long-lasting anti-tumor immunity, as well as imaging and thermal enhancement.

### 5.4 Clinical studies undertaken

In the past, a large number of clinical trials have confirmed the anti-tumor effect of natural polysaccharides (**Table 2**). However, whether this effect is related to the fact that natural polysaccharides enhance the activity and function of DC has not been verified in humans. In recent years, clinical trials of natural polysaccharides acting on DCs to exert anti-tumor activity are being conducted.

**Table 2:** Clinical trial of natural polysaccharides against tumor.

Polysaccharide type	Whether to combine with other therapies	Cancer specie	Intervention time	Result	References
Coriolus versicolor polysaccharide (CVP)	No	Advanced non-small cell lung cancer	28 days	After 28-day treatment, there was a significant improvement in blood leukocyte and neutrophil counts, serum IgG and IgM, and percent of body fat among the treatment group	(143)
Grifola frondosa polysaccharide	No	Breast cancer	3 weeks	The intermediate dose (5–7 mg/kg per day) was associated with the most prominent functional changes, such as increased production of IL-2, IL-10, TNF-α and IFN-γ by subsets of T cells	(144)
GLP	No	Advanced colorectal cancer	12 weeks	GLP tended to increase counts of CD3, CD4, CD8 and CD56 lymphocytes, plasma concentrations of IL-2, IL-6 and IFN-γ, and NK activity	(145)
APS	Chemotherapy	Advanced non-small cell lung cancer	3 months	APS prolonged the median survival time of patients and improved fatigue, nausea and vomiting, pain, loss of appetite and other symptoms	(146)
GLP	No	Advanced-stage cancer	12 weeks	After 12 weeks of treatment, the mean serum concentrations of IL-2, IL-6, and IFN-γ increased significantly, the mean absolute number of CD56+ cells as well as NK cell activity increased significantly, and the number of CD3+, CD4+, and CD8+ cells increased slightly compared to baseline levels	(147)
CVP	No	Liver cancer	12 weeks	Coriolus versicolor polysaccharide subjects had less appetite loss and pain symptoms compared to placebo subjects during treatment	(148)
LNT	No	Advanced prostate carcinoma	3 months	The 50% survival length of treated and control patients was 48 and 35 months, respectively	(149)
LBP	Immunotherapy	Advanced cancer	3 months	LAK/IL-2 plus LBP treatment led to more marked increase in NK and LAK cell activity than LAK/IL-2 without LBP	(150)

MGN-3 is a plant-derived arabinoxylan. Cholujova et al. (151) conducted a randomised, placebo-controlled study in 48 patients with multiple myeloma, who received 2 g of MGN-3 granule powder or an equivalent amount of placebo dissolved in water per day. A significant increase in myeloid DC (MDC) levels in peripheral blood was found after treatment (25.8 ± 3.6% vs. 17.6 ± 2.6%), in addition to an increase in NK lysogenic activity (56.6 ± 12.2 LU vs. 30.8 ± 7.4 LU). Loveland et al. (152) conducted a phase I clinical trial in which the investigators pulsed DCs with mannan-MUC1 fusion protein (MFP) and found that MFP increased the expression of the molecules CD40, CD83, and CD86 on the surface of the DCs. Afterwards, the investigators intradermally injected the stimulated DCs into the upper arms or legs of 10 patients with different types of tumors and followed up with all the patients, finding that 4 patients had stable disease over the 12-week period of the study, and 2 patients had stable disease over the 3-year period of the study. Ma et al. (153) used intradermal injection of autologous DCs combined with intravenous infusion of ginseng polysaccharide (GPS) to treat 96 patients with non-small cell lung cancer, and found that Th1 (INF-γ, IL-2) and Th1/Th2 (INF-γ/IL-4, IL-2/IL-5) were higher than the control in the study group and lower than the control group in the Th2 (IL-4, IL-5) after the treatment, which indicated that GPS was able to maintain the dynamic balance of Th1/Th2. In addition, GPS significantly prolonged the progression-free survival of lung cancer patients.

## 6 Trends and recommendations

### 6.1 The combined application of polysaccharides

Studies have shown that fungal polysaccharides appear to have a better anti-tumor capacity, especially in endocrine-related tumors, and this effect depends on the β-glucans with DB 0.2-0.33, MW of 100-200 kDa, and a triple-helix structure (154). Compared to other polysaccharides, plant polysaccharides possess the strongest immune-enhancing effect, which is related to their 1→3, 6 branched galactose residues, the rhamnoides galacturonic acid, and other functional groups (155).

Kikete et al. (156) assessed the immune modifying activity of APS and CP, as well as a mixture of these two polysaccharides to activate DCs loaded with tumor cell lysate. They found that of the three groups of DCs, the APS-treated group resulted in the most significant increase in CD40 expression, while the CP group had the most upregulated expression of CD80 and CD86. The phenomenon may be due to differences in the monosaccharide residues contained by the two polysaccharides. Although both are rich in glucose with little or no mannose residues, CP contains more galactose than APS. When used as a DC vaccine adjuvant, pro-inflammatory cytokines such as IL-6, G-CSF, CCL3, CCL1, and IL-1β increased 4-7 fold in the treatment group with CP and APS alone, and surprisingly, 7-15 fold in the combined APS+CP group. It can be seen that the combined application of the two has complementary advantages.

Therefore, combining two or more polysaccharides may produce a stronger synergistic effect of enhancing DCs function relative to each other. However, further research is required to determine the combination that will provide the highest potency.

### 6.2 Structural modifications of polysaccharides

Not only the source of extraction of polysaccharides but also the higher spatial conformation, glycosidic bonding mode, and functional group modification of polysaccharides are essential factors affecting the pharmacological activity of polysaccharides (157). Under chemical modification modes, such as carboxymethylation, sulphation, phosphorylation and selenization, certain functional groups are able to be replaced or introduced into polysaccharides under specific conditions, thus altering the molecular weight, structure, position, and number of substituents of polysaccharides. By changing these parameters, the physicochemical and functional properties of polysaccharides are also altered (158).

Qin et al. (159) used the HNO3-Na2SeO3 method to selenically modify the Hericium erinaceus polysaccharide (HEP) and investigated the effects of nine selenium derivatives, sHEP1-sHEP9, on the phenotypic and functional maturation of mouse BMDCs. The results showed that both sHEP2 and sHEP8 significantly increased the expression of the surface molecules MHC-II and CD86 and decreased the uptake of FITC-Dextran by DCs, compared to unmodified HEP as a control. In particular, sHEP2, which had the highest effector intensity, also increased phosphorylation of p38, ERK, and JNK, as well as degradation of IκB-α/β and nuclear translocation of p65 and p50, suggesting that sHEP2 can promote DCs maturation and activation by enhancing MAPK and NF-κB signalling downstream of TLR4. Wang et al. (160) divided the Carboxymethylated plantago polysaccharide (CM-PLCP) into five gradients (0.40-0.62) and tested the immunomodulatory activity of CM-PLCP on DCs *in vitro*. Compared with unmodified PLCP, DCs treated with CM-PLCP and MHCII, CD86 and CD80 expressed higher levels of surface molecules. The secretion of IL-12p70 was increased, the mRNA expressions of CCR7 and CXCR4 chemokines were increased, and the endocytosis activity was inhibited. It can be concluded that carboxymethylated polysaccharide as an immunotherapy adjuvant has a wide prospect in the treatment of cancer. Feng et al. (161) injected phosphorylated Radix Cyathulae officinalis Kuan polysaccharides (pRCPS) into the mouse subcutaneously, and removed the spleen 14 days later. The results showed that the expression levels of MHC-II, CD80, CD40 and CD86 on DCs in spleen of mice in pRCPS group were significantly higher than those in other groups, indicating that pRCPS could significantly enhance humoral and cellular immune responses by inducing DCs maturation. Liu et al. (162) fed mice with different concentrations of Porphyra haitanensis polysaccharide (PHPS) and found that the number of MHC-II+CD11c+DCs in the spleens of mice in the PHPS 50, 150, and 250 mg/kg groups were increased to varying degrees (6.92%, 10.69%, and 9.78%), in addition to significantly higher levels of TNF-α and IL-10.

In summary, further studies on the complete molecular weight range, monosaccharide species, linkage patterns, and ionic properties of polysaccharides that exert biological activity, clarification of structure-activity relationships, and chemical modification by introducing new groups into polysaccharides are potential ways to improve the immunostimulatory activity of polysaccharides (163).

### 6.3 Validation of *in vivo* studies

Although the pro-DCs activation and anti-tumor activity of polysaccharides have been verified *in vitro* studies, the biological effect of polysaccharides in the human body is related to serum polysaccharide concentration. Pang et al. (164) found that the recommended dosages of LNT used clinically were 1mg, 4mg, and 8mg, and pharmacokinetic studies showed that when 1mg LNT entered the human body after absorption and metabolism, the blood concentration was only 1.7 μg/ml, and only when the blood concentration exceeded 6.8 μg/ml could it effectively activate immune cells such as DCs and exert anti-tumor effects.

In spite of the fact that polysaccharides are less toxic than other immunostimulatory drugs, the adverse effects of their action on the body cannot be ignored. The glycoproteins in the polysaccharide product are fully antigenic when they enter the body and are covalently bonded to macromolecules. Impurities that are not completely removed during the extraction process can also stimulate mast cells and basophils, mediating the release of histamine, bradykinin, prostaglandins, and chemokines into the blood and tissues, triggering type I hypersensitivity reactions such as rashes and, in severe cases, anaphylaxis may occur (165). In addition to allergic reactions, musculoskeletal system damage such as joint soreness, back pain, and lumbar spine pain may also appear 30 minutes after the first dose, and acute asthma and cardiac arrest have been reported with LNT injection (166).

Therefore, relevant physiological or pharmacological concentrations should be used in *in vitro* studies, appropriate *in vivo* simulation systems should be developed to verify the pharmacokinetic characteristics of polysaccharides, and extensive sample preclinical studies and clinical studies should be carried out to observe the safety of polysaccharide acting in humans, so as to provide the pharmacological and clinical basis for the application of polysaccharides.

## 7 Conclusion

Polysaccharides offer considerable safety, relative affordability, and powerful immunomodulatory functions, providing a new research direction for tumor immunotherapy. This paper reviews the mechanisms and applications of polysaccharides to promote specific cellular and humoral immunity by acting on DCs, then kill tumor cells. According to current studies, polysaccharides act on PRRs and promote the maturation of DCs through various pathways such as PI3K/AKT, MAPK, NF-κB, and Dectin-1/Syk, specifically by increasing the expression of costimulatory molecules and MHC class molecules, weakening endocytosis, activating T cells, and increasing the secretion of pro-inflammatory cytokines, in addition to regulating the intracellular metabolism of DCs, thereby enhancing immune response and playing an important role in tumor invasion and improvement of tumor disease prognosis. In view of the above advantages, polysaccharides have been used as adjuvants in the preparation of DC vaccines, as well as in combination with DC-CIK cells and ICIs for immunotherapy, and delivery systems made by encapsulating polysaccharides in NPs also avoid the rapid metabolism of polysaccharides and enhance the antigenic stimulation of vaccines.

However, more mechanistic studies are needed to elucidate the molecular basis of the regulatory effects of polysaccharides on DCs. Due to the heterogeneity of DCs, future studies should further explore the effects of polysaccharides on specific DC subpopulations. In addition, although some progress has been made in laboratory research, using polysaccharides in clinical settings still faces various challenges. The primary obstacles, such as poor oral bioavailability and difficulty in targeting organs, need to be addressed. The minimum active concentration of polysaccharides as adjuvants also needs to be studied. The pharmacokinetics of polysaccharides in the intestinal tract and blood should be further studied, and structural modification should be carried out according to the effects of polysaccharides. Finally, we also ought to carry out more rigorous clinical trials to obtain sufficient data to support the effectiveness and safety of polysaccharides activated DCs, so as to promote the clinical application of natural polysaccharides.

## Author contributions

HX: Writing – original draft. XH: Writing – review & editing. LC: Writing – original draft. HZ: Writing – review & editing.

## Glossary

**Table:**

**ABPS**	Achyranthes bidentata polysaccharide
**ACP**	Acid phosphatase
**AKT**	protein kinase B
**APC**	antigen presenting cell
**APS**	Astragalus polysaccharide
**BMDC**	bone marrow-derived DC
**cDC1**	type 1 classical DC
**cDC2**	type 2 classical DC
**CDNs**	cyclic dinucleotides
**CDP**	Common DC progenitor
**cGAS**	cyclic guanosine monophosphateadenosine monophosphate synthase
**CIK**	Cytokine-induced killer
**CLR**	C-type lectin receptor
**CP**	Codonopsis polysaccharide
**CTL**	Cytotoxic T lymphocyte
**CTLA-4**	Cytotoxic T-lymphocyte antigen-4
**CVP**	Coriolus versicolor polysaccharide
**CXCL9**	C-X-C motif chemokine ligand 9
**DAMP**	damage associated molecular pattern
**DC**	Dendritic cell
**DOX**	doxorubicin
**FLT3L**	Fms-like tyrosine kinase 3 ligand
**GLP**	Ganoderma lucidum polysaccharide
**GMDP**	DC progenitor
**GPS**	Ginseng polysaccharide
**GPX4**	glutathione peroxidase 4
**HEP**	Hericium erinaceus polysaccharide
**HIF**	Hypoxia-inducing factor
**HMGB1**	high mobility group protein B1
**HSC**	Hematopoietic stem cell
**ICIs**	Immune checkpoint inhibitors
**iNOS**	Inducible nitric oxide synthase
**LBP**	Lycium barbarum polysaccharide
**LC**	Langerhans cell
**LNT**	lentinan
**MAPK**	mitogen-activated protein kinase
**M-CSF-R**	macrophage colony stimulating factor receptor
**MDP**	monocyte and DC progenitor
**MFP**	mannan-MUC1 fusion protein
**MHC**	Major histocompatibility complex
**moDC**	monocyte-derived DC
**MyD88**	myeloid differentiation factor 88
**NF-κB**	nuclear factor-κB
**NGP**	Neutral Ginseng polysaccharide
**NK**	natural killer
**NP**	nanoparticle
**PAMP**	pathogen associated molecular pattern
**PBMC**	peripheral blood mononuclear cell
**pDC**	plasmacytoid DC
**PD-1**	programmed cell death protein 1
**PD-L1**	programmed cell death-ligand 1
**PFPS**	Pleurotus ferulae polysaccharide
**PHPS**	Porphyra haitanensis polysaccharide
**PI3K**	phosphatidylinositol 3-kinase
**PLGA**	Poly (D,L-lactide-co-glycolide)
**PLP**	Plantain polysaccharide
**POL-P3b**	Portulaca oleracea L. polysaccharide
**pre-cDC**	classical DC precursor
**pre-pDC**	plasmacytoid DC precursor
**PRR**	Pattern recognition receptor
**RGP**	Rehmannia glutinosa polysaccharide
**ROS**	Reactive Oxygen Species
**STING**	stimulator of interferon genes
**TAA**	tumor-associated antigen
**Th1**	type 1 helper T
**TLR**	Toll-like receptor
**TME**	tumor microenvironment
**VEGF**	Vascular endothelial growth factor
